# Supplementary material for: Development and validation of an obstetric early warning system model for use in low resource settings
Source: BMC Pregnancy Childbirth. 2020 Sep 11;20:531. doi: 10.1186/s12884-020-03215-0 (PMC7488502; doi:10.1186/s12884-020-03215-0)
Supplement: Supplementary file 1 — Additional file 1. CEMACH MEOWS. [file 12884_2020_3215_MOESM1_ESM.pdf]

APPENDIX 1: CEMACH MEOWS

Name:	Ward:	Consultant:
Hospital Number:	Date of Birth:	Height:

Date:																		
Time:																		

Temperature °C

39																		39
38																		38
37																		37
36																		36
35																		35

Individual Parameters >37°
....

Systolic blood pressure

200																		200
190																		190
180																		180
170																		170
160																		160
150																		150
140																		140
130																		130
120																		120
110																		110
100																		100
90																		90
80																		80
70																		70
60																		60
50																		50
MAP mmHg																		

Individual Parameters SBP>
....

Diastolic blood pressure

130																		130
120																		120
110																		110
100																		100
90																		90
80																		80
70																		70
60																		60
50																		50
40																		40

Individual Parameters DBP:
....

Heart rate

170																		170
160																		160
150																		150
140																		140
130																		130
120																		120
110																		110
100																		100
90																		90
80																		80
70																		70
60																		60
50																		50
40																		40

Individual Parameters Pulse Rate:
....

RESP (write rate in corresp. box)	>30																		>30
	21–30																		21–30
	11–20																		11–20
	0–10																		0–10
Saturations	95–100%																		11–20
	<95%																		95–100%
Administered O_2 (L/min.)																			%

Individual Parameters Resp. Rate:
....

Date:																		
Time:																		

URINE	passed (Y/N)																		passed (Y/N)
Proteinuria	protein ++																		protein ++
	protein >++																		protein >++
Urinalysis																			
Oedema (Y/N)																			
Amniotic fluid	Clear/Pink																		Clear/Pink
	Green																		Green
NEURO RESPONSE (✔)	Alert																		Alert
	Voice																		Voice
	Pain																		Pain
	Unresponsive																		Unresponsive
Pain Score (no.)	0–1																		0–1
	2–3																		2–3
Lochia	Normal																		Normal
	Heavy/Fresh/ Offensive																		Heavy/Fresh/ Offensive
Looks unwell	NO (✔)																		NO (✔)
	YES (✔)																		YES (✔)
Trigger	NO (✔)																		NO (✔)
	YES (✔)																		YES (✔)

Wound site check																	
Blood glucose																	
Nausea score																	
Bowel action																	
Daily weight																	

**Neuro Responses**	
Alert	Patient is alert and concious
Verbal	Patient responds to verbal stimulus
Pain	Patient responds to painfull stimulus
Unresponsive	Patient is unresponsive to any stimulus

PAIN SCORE (assess pain on movement, deep breathing or coughing)

No pain at rest or on movement	0
No pain at rest, slight pain on movement	1
Intermittent pain at rest, moderate pain on movement	2
Intermittent pain at rest, moderate pain on movement	3

NAUSEA SCORE

None	0
Nausea	1
Vomiting	2
