# Supplementary material for: Development and validation of an obstetric early warning system model for use in low resource settings
Source: BMC Pregnancy Childbirth. 2020 Sep 11;20:531. doi: 10.1186/s12884-020-03215-0 (PMC7488502; doi:10.1186/s12884-020-03215-0)
Supplement: Supplementary file 2 — Additional file 2. Obstetric early warning chart [file 12884_2020_3215_MOESM2_ESM.pdf]

OBSTETRIC EARLY WARNING CHART

Name: ________________________ Hosp number: ____________

Ward: ________________________ Consultant: ______________

Diagnosis______________________ Gravidity/Parity: __________

For score of 0 or 1, repeat observations 12 hourly or as usual for post op patients
For score of 2, repeat after 30 minutes, if it remains 2 or rises, inform Doctors
For score of 3 or more, or if concerned regardless of the score, please call doctors
Doctors review time should be completed for triggered patients

Date	Time		Temp	Pulse	RR	Sys BP	Urine	Cons level	Del mode	EWS	Review time	Signature
		Observation										
		Score										
		Observation										
		Score										
		Observation										
		Score										
		Observation										
		Score										
		Observation										
		Score										
		Observation										
		Score										
		Observation										
		Score										
		Observation										
		Score										
		Observation										
		Score										

Score	2	1	0	1	2
**Temperature**	<35	35 – <36	36 – <38		>38
**Pulse rate**	<40	40 – <50	50 – <100	100 – 120	>120
**Respiratory rate**	0 – 10		11 – 20	21 – 30	>30
**Systolic BP**	<90	90 – <100	100 – <150	150 – 160	>160

Score	0	1	2
**Urine (ml/hr)**	>30	20 – 30	<20
**Conscious level (AVPU)**	Alert	Response to voice	Response to pain/unresponsive
**Delivery mode**	SVD	C/S	
