# Supplementary material for: Development and validation of an obstetric early warning system model for use in low resource settings
Source: BMC Pregnancy Childbirth. 2020 Sep 11;20:531. doi: 10.1186/s12884-020-03215-0 (PMC7488502; doi:10.1186/s12884-020-03215-0)

**Appendix 4:** Receiver-operating curve for the prediction of severe maternal outcome in the validation model (n = 900)


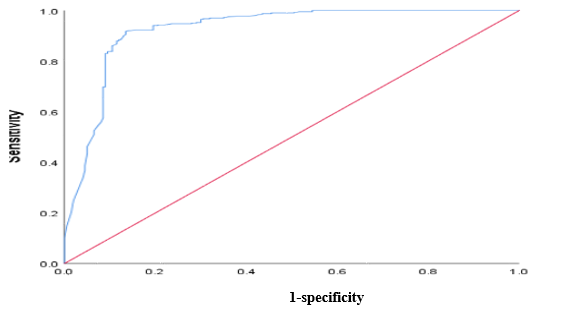

Supplement: Supplementary file 4 — Additional file 4. Receiver-operating curve for the prediction of severe maternal outcome in the validation model (n = 900) [file 12884_2020_3215_MOESM4_ESM.docx]
