# Supplementary material for: Development and validation of an obstetric early warning system model for use in low resource settings
Source: BMC Pregnancy Childbirth. 2020 Sep 11;20:531. doi: 10.1186/s12884-020-03215-0 (PMC7488502; doi:10.1186/s12884-020-03215-0)
Supplement: Supplementary file 5 — Additional file 5. Receiver-operating curve for the prediction of severe maternal outcome in the sensitivity model with total dataset (n = 5243) [file 12884_2020_3215_MOESM5_ESM.docx]

**Appendix 5:** Receiver-operating curve for the prediction of severe maternal outcome in the sensitivity model with total dataset **(n=5243)**


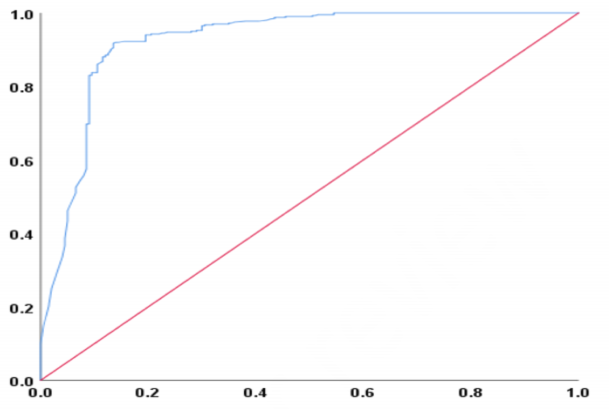


**1-Specificity**

**Sensitivity**
